# Stem-Cell-Derived Extracellular Vesicles: Unlocking New Possibilities for Treating Diminished Ovarian Reserve and Premature Ovarian Insufficiency

**DOI:** 10.3390/life13122247

**Published:** 2023-11-23

**Authors:** Yana O. Martirosyan, Denis N. Silachev, Tatiana A. Nazarenko, Almina M. Birukova, Polina A. Vishnyakova, Gennadiy T. Sukhikh

**Affiliations:** 1 V.I. Kulakov National Medical Research Center for Obstetrics Gynecology and Perinatology, Ministry of Healthcare of Russian Federation, 117997 Moscow, Russia; t_nazarenko@oparina4.ru (T.A.N.); a_birukova@oparina4.ru (A.M.B.); p_vishnyakova@oparina4.ru (P.A.V.); g_sukhikh@oparina4.ru (G.T.S.); 2 Department of Functional Biochemistry of Biopolymers, A.N. Belozersky Research Institute of Physico-Chemical Biology, Moscow State University, 119992 Moscow, Russia; 3 Research Institute of Molecular and Cellular Medicine, Peoples’ Friendship University of Russia (RUDN University), 117198 Moscow, Russia

**Keywords:** mesenchymal stem cells, microvesicles, premature ovarian insufficiency, angiogenesis, extracellular vesicles

## Abstract

Despite advancements in assisted reproductive technology (ART), achieving successful pregnancy rates remains challenging. Diminished ovarian reserve and premature ovarian insufficiency hinder IVF success—about 20% of in vitro fertilization (IVF) patients face a poor prognosis due to a low response, leading to higher cancellations and reduced birth rates. In an attempt to address the issue of premature ovarian insufficiency (POI), we conducted systematic PubMed and Web of Science research, using keywords “stem cells”, “extracellular vesicles”, “premature ovarian insufficiency”, “diminished ovarian reserve” and “exosomes”. Amid the complex ovarian dynamics and challenges like POI, stem cell therapy and particularly the use of extracellular vesicles (EVs), a great potential is shown. EVs trigger paracrine mechanisms via microRNAs and bioactive molecules, suppressing apoptosis, stimulating angiogenesis and activating latent regenerative potential. Key microRNAs influence estrogen secretion, proliferation and apoptosis resistance. Extracellular vesicles present a lot of possibilities for treating infertility, and understanding their molecular mechanisms is crucial for maximizing EVs’ therapeutic potential in addressing ovarian disorders and promoting reproductive health.

## 1. Introduction

In a recent report published by the World Health Organization (WHO) in 2022, the global prevalence of infertility is estimated at 17.5%, with the highest rates in the Western Pacific region, reaching a peak of 23.2% [1]. Despite significant advances in assisted reproductive technology (ART), achieving successful pregnancy rates remains a challenge. The success rate is only around 37% per cycle, and this rate continues to decrease as the mother ages [2]. In an effort to improve cycle success, healthcare providers often offer additional technologies to standard procedures: for example, the use of stem cell therapy in the treatment of Asherman syndrome and the use of platelet-rich plasma (PRP) or PRP in combination with minimally manipulated endometrial cells to treat a refractory thin endometrium [3,4,5,6,7].

A major obstacle contributing to the decreased efficacy of in vitro fertilization (IVF) programs is the prevalence of diminished ovarian reserve (DOR) and premature ovarian insufficiency (POI, or premature ovarian failure—POF) in infertile patients. In addition, the age-related decline in follicle number leads to a lower availability of oocytes in IVF cycles [8] in the poor prognosis group characterized by a poor response to ovarian stimulation, resulting in a higher dropout rate of ART cycles and a lower number of live births. The major limitation to the effectiveness of IVF programs is the limited ability to significantly influence the number and competence of oocyte–cumulus complexes obtained.

POI is a multifaceted condition affecting 1% of women of a reproductive age. It is characterized by elevated gonadotropin levels, including follicle-stimulating hormone (FSH), and irregular or absent menstruation before the age of 40 [9]. Another condition, DOR, also known as a poor ovarian response to stimulation, is also quite heterogeneous in nature and is commonly seen in fertility treatments in regularly menstruating women [10]. It is worth noting that many cases of DOR, observed in clinical practice, are related to physiological factors and represent an age-related decline in ovarian reserve.

Clinical guidelines have been established worldwide for predicting the ovarian response to stimulation and diagnosing a decline in ovarian reserve. According to the clinical guidelines of the European Society of Human Reproduction and Embryology (ESHRE), ovarian reserve is preferably assessed using indicators such as the number of antral follicles or the level of anti-Muellerian hormone (AMH). According to the Bologna criteria, an AMH level below 1.1 ng/mL is an indication that the patient may have a potential poor ovarian response [11]. Meanwhile, the American College of Obstetricians and Gynecologists and the American Society of Reproductive Medicine suggest that an AMH level below 1 ng/mL and an FSH level above 10 mIU/mL indicate DOR [12,13,14].

To improve the results of ovarian stimulation, researchers have explored a number of strategies. These include switching to higher doses of gonadotropins, altering ovarian stimulation protocols, using “freeze-all” strategies and various other approaches [15]. However, these approaches raise concerns about potential adverse effects on oocyte quality and the differential impact of different protocols on ART outcomes in different cycles [16]. The proposed methods for ovarian stimulation in patients with DOR include a range of strategies, including delayed-onset gonadotropin-releasing hormone (GnRH) agonists [17,18], microdose GnRH agonist [19,20,21], multiple-dose GnRH antagonist [22,23,24], mild ovarian stimulation [25], long GnRH agonist [26,27,28], GnRH antagonist [29,30,31], GnRH antagonist/letrozole [32,33], progestin-primed ovarian stimulation [34,35], short GnRH agonist [36,37], duo-stim and random-start ovarian stimulation protocols [11]. According to Di et al. [38], among these treatment regimens, the delayed-start GnRH agonist and the microdosed GnRH agonist have emerged as the two most promising approaches for the treatment of DOR and show favorable clinical outcomes.

It is evident that a change in gonadotropin regimens, where drug administration does not begin until day 2 of the menstrual cycle, when follicles have entered the gonadotropin-dependent growth phase, affects a pre-programmed and established follicular pool [39]. The fate of these follicles is determined 90–120 days before the start of the IVF protocol, emphasizing the crucial role of the gonadotropin-independent phase of follicular development in shaping the fate of future oocytes and embryos [40]. Therefore, there are compelling reasons to explore strategies that target primordial follicles before they enter the gonadotropin-dependent stage of development [41,42,43,44].

The prospect of reactivating dormant follicles in the ovarian cortex has attracted considerable attention after Li et al. demonstrated their reactivation and successful development into healthy offspring using an in vitro activation (IVA) (protocol [45]). In this innovative approach, PTEN (phosphatase and tensin homolog) inhibitors and PI3K (phosphatidylinositol 3-kinase) stimulators, in particular AKT (serine/threonine protein kinase 1) stimulators, were used. The work of Li et al. is a fundamental achievement in ovarian reactivation techniques. In addition, studies of ovarian biopsies from women with POI have shown that nearly 40% of these women retained some (30%) or many (9%) follicles in the ovarian cortex [46].

Although mammalian ovaries harbor numerous follicles, the majority of them remain dormant for long periods of time, even decades. In 2004, Tilly’s research challenged the conventional assumption of a limited number of oocytes in female mammals by identifying ovarian stem cells in the ovarian cortex [47]. Subsequent studies in mice and other mammals, including humans, confirmed these findings [43,44,48,49,50,51,52]. Nevertheless, the data from these studies have been repeatedly questioned [53]. Currently, two types of ovarian stem cells have been identified: pluripotent cells with a diameter of 2–4 μm, called Very Small Embryonic-Like Stem Cells (VSELs), and the ovarian stem cells themselves, which have a diameter of 5–8 μm and express the cytoplasmic isoform of the transcription factor Oct-4B. Among the markers, there are both common and typical markers of stem cells, such as Oct-4, Nanog and SOX-2, and more specific ones, such as STRO-1, FRAGILIS, LIN28, etc. It is likely that stimulation of these cells is one of the possible mechanisms underlying such a method of activating the ovarian reserve as stem cell transplantation. It is unclear how exactly mesenchymal stem cells improve ovarian function. Extracellular vesicles (EVs), a critical component in the new paradigm of intercellular communication, enable transplanted stem cells to communicate with cellular components of ovarian tissue and secrete pro-regenerative substances. Despite the accumulated knowledge on the mechanisms of action of EVs, this issue has not yet been adequately addressed in diseases such as POI and DOR, which was the main motivation for the writing of this review.

A systematic literature search was conducted until 21 June 2023 in the PubMed and Web of Science databases using the keywords “((stem cells) AND (extracellular vesicles)) AND (exosomes)” (first search strategy) and “(stem cells) AND (exosomes)” AND (premature ovarian insufficiency) AND “diminished ovarian reserve” (second search strategy). Only articles found with both strategies and in both databases were included. Articles were excluded if the article was written in any language other than English or Russian; they were duplicates; they were reviews, commentaries, thesis, book chapters; the terms exosomes or EVs did not occur. In the first phase, the articles from both databases were screened for eligibility based on the title and abstracts. Subsequently, all full-text articles that had been checked for eligibility were evaluated for inclusion in the study.

## 2. Premature Ovarian Insufficiency

The ovarian reserve represents the cumulative number of follicles in the ovaries. It includes both nongrowing follicles and follicles that are recruited at various stages of growth, from preantral to antral, to ovulation. According to the widely accepted theory, women are endowed with a limited number of ovarian follicles at birth. This number declines markedly during intrauterine development, dropping from a peak of about 7 million to 1 million at the time of birth. This decline continues through childhood, with about 400,000 follicles remaining at the onset of menarche. Eventually, as menopause approaches, fewer than 1000 follicles remain in the ovaries [54,55].

Furthermore, this gradual decline in the number of follicles with age is accompanied by a sequence of reproductive events. This sequence begins with a decline in fertility, progresses to the natural onset of infertility and often includes disruptions of the menstrual cycle that eventually lead to the complete absence of menstruation at menopause. Theoretically, these phases develop over “fixed time intervals” that precede the transition to the next phase [56,57]. Consequently, three different scenarios may manifest: a “normal” age-related decline in ovarian reserve, a DOR, due to prenatal factors, or a decline in ovarian reserve resulting from the influence of adverse environmental factors or nutritional deficiencies [58].

The etiology of an early loss of ovarian reserve is associated with several factors, including iatrogenic influences, autoimmune factors, chromosome X-associated abnormalities and point gene mutations [48]. In particular, iatrogenic causes such as ovarian surgery, gonadotoxic therapies and certain medical treatments contribute significantly to the development of POI and decreased ovarian reserve [48]. Of the various iatrogenic factors contributing to POI, a significant proportion is attributed to ovarian surgery, which accounts for approximately 64% (excluding bilateral ovariectomy) [48]. In addition, radiotherapy and chemotherapy used in the treatment of a variety of diseases, including both malignant and benign conditions, are also common causes of POI [48,59]. Mutations and decreased expression of critical genes related to DNA repair and mitochondrial function may accelerate the depletion of follicular reserve in POI and middle-aged women, respectively. Whereas the process of follicle formation was previously thought to be predominantly under the direct control of the central nervous system, current understanding emphasizes the essential role that paracrine mechanisms play in its regulation.

The decrease in ovarian follicular reserve is due to the periodic, sequential activation of primordial follicles coming out of dormancy. Primordial follicle activation is a crucial step in folliculogenesis that ultimately culminates in the selection of a single oocyte for ovulation. An abnormal acceleration of this activation process can significantly reduce the ovarian reserve [60]. Currently, depletion of POI is thought to be caused by excessive acceleration of activation of the pool of primordial follicles. Maintaining a high ovarian reserve depends on a balance between activating and inhibitory factors. Recent studies have focused on PTEN/PI3K/AKT/Forkhead Box O3 (FOXO3) and the Hippo signaling pathway [61], which will be discussed in more detail below.

Modern strategies for controlled ovarian stimulation primarily target antral follicle growth, whereas dormant primordial follicles do not respond to conventional stimulation protocols. Understanding the biological basis of primordial follicle activation is extremely important but has been relatively elusive. Primordial follicle activation is a complex interplay of various local factors and intracellular signaling pathways. Activators such as BMP4/7, GDF-9, KIT ligand, FGF2/7, insulin, GREM1/2 and LIF and suppressors including AMH, LHX8, PTEN, Tsc1m/TORC1, FOXO3a, YAP/Hippo signaling pathway and FOXL2 have been associated with primordial follicle activation [62,63]. Most of the knowledge about signaling networks and molecules involved in primordial follicle activation has been obtained from rodent premature ovarian failure models [64,65].

Although the complete mechanism of primordial follicle activation is not yet fully understood, studies conducted on murine models have shown that specific deletion of the PTEN and FOXO3 genes in oocytes promotes the activation and growth of all primordial follicles [66,67]. The PTEN gene encodes a phosphatase enzyme that negatively regulates the PI3K/AKT/FOXO3a signaling cascade [68,69]. Stimulation of dormant primordial follicles has also been achieved with the use of PTEN inhibitors and AKT activators in both mice and humans. In the ovaries, the AKT protein plays an important role within the PI3K/AKT/mTOR signaling pathway and is expressed by both oocytes and granulosa cells of human follicles [61,69]. AKT targets a broad spectrum of molecules that directly and indirectly affect follicle activation [68,70]. This signaling pathway includes a variety of regulatory molecules that exert inhibitory control within the kinase cascade. Ultimately, this cascade counteracts the transcriptional coactivator Yes-associated protein (YAP) together with its PDZ-binding motif (TAZ), thereby suppressing cell growth [61]. Maintaining the balance between cell proliferation and apoptosis is necessary to maintain organ size and tissue homeostasis throughout life. In mammals, both processes are coordinated via the Salvador–Warts–Hippo signaling pathway.

Numerous genetic factors have been associated with POI, with certain genes such as GDF-9 (growth differentiation factor 9), BMP-15 (bone morphogenic protein 15) [49], FOXL2 [50], FSHR (follicle-stimulating hormone receptor) [51], STAG3 (stromal tumor antigen 3) [52], XRCC2 (X-ray repair cross-compliment 2) [53], MCM8 [71], NRIP1, XPO1 and MACF1 [72] showing significant associations. The impairment of primordial follicle activation emerges as a central biological mechanism underlying premature loss of ovarian reserve, regardless of etiology [73,74]. Animal models, often involving chemotherapeutic agents, mental stress, galactosemia, ovarian antigen peptides, autoimmunity activation (pellucid glycogen ZP3) or genetically manipulated mice, have shed light on the intricate mechanisms behind follicular apoptosis and autophagy inhibition during follicle maturation. However, there may be a number of adverse consequences, such as myelosuppression in the chemotherapy-induced POI or low stability of autoimmunity or mental stress in the POI animal model. The analysis of existing animal models and the POI therapies carried out with them shows that the most effective treatment methods are hormone replacement therapy and stem cell transplantation.

Disturbances in this delicate balance between cell proliferation and apoptosis in the ovaries, as evidenced by the premature activation and rapid depletion of follicles in AMH-knockout mouse models, underscore the importance of ovarian homeostasis in maintaining the follicular pool. It is widely accepted that AMH regulates the amount of growing follicles and influences their selection for ovulation [75]. AMH primarily exerts a negative regulatory role especially in the early stages of follicular development [76]. It accomplishes this by suppressing both the recruitment and growth of follicles, effectively dampening the actions of growth factors and gonadotropins, particularly FSH [77]. Experimental studies have shown that the number of growing follicles decreases by about 40–50% when AMH is introduced into cultured human ovarian cortical tissue [78]. This highlights the ability of AMH to inhibit the initial stages of follicle development, especially those dependent on FSH. Notably, the absence of AMH leads to a more rapid depletion of the ovarian follicular pool [79]. In genetically modified female mice with AMH knockout, ovulation ceases at about 16–17 months of age, whereas older wild-type female mice continue to have normal menstrual cycles [80]. At 13 months of age, the pool of primordial follicles decreases three-fold in female mice lacking AMH compared to wild-type mice. AMH also affects estrogen biosynthesis by inhibiting aromatase activity, resulting in a decrease in estrogen production [81]. It is noteworthy that AMH concentration in the follicular fluid of patients undergoing in vitro fertilization correlates with estradiol concentration, suggesting a possible role of AMH as a coregulator of steroidogenesis [75]. In addition, AMH plays an autocrine role in the maturation of normal follicles. Studies in mice have shown that AMH inhibits the first meiotic division of diplotene oocytes [82]. In human granulosa cells, AMH also shows its influence by blocking proliferation in vitro [83]. Oocytes within a pool of growing follicles may exert control over a pool of primordial follicles by modulating the expression of AMH [76]. Age-related alterations in AMH levels contribute to accelerated follicular activation, which in turn contributes to the overall process of ovarian reserve loss [84].

Numerous preclinical and clinical studies have shown that the field of stem-cell-based therapeutics is very promising and has attracted significant interest due to its potential to treat POI [85,86]. It is noteworthy that even in cases where the ovaries lose their ability to ovulate, a reserve of dormant follicles remains, offering the possibility of growth under the influence of stem cells [87,88]. Consequently, further research and development in this field may pave the way for revolutionary therapies in the future.

Results regarding anti-age-associated ovarian hypofunction effects and improvement of ovarian function have been shown after mesenchymal stem cell transplantations in mouse models [89,90]. One of the first papers to use this method in humans was the study by Herraiz and colleagues [91], in which autologous ovarian stem cell transplantation (ASCOT) significantly improved ovarian function by increasing the number of antral follicles and oocytes in 81.3% of patients who responded poorly. Although the embryo euploidy rate was low, ASCOT managed to overcome previous limitations and enable pregnancy via enhancement of existing follicles. A number of papers show encouraging results of this technology, which are well described in the review by Fàbregues and colleagues [92]. Exactly how mesenchymal stem cells improve ovarian function is not entirely clear. In addition to the secretion of pro-regenerative factors, transplanted stem cells may interact with the cellular components of ovarian tissue using EVs.

## 3. Effects of Stem Cell Therapy

### 3.1. Transplantation on Ovarian Follicles and Function

Thanks to their multipotent differentiation abilities and paracrine properties, multipotent MSCs have established themselves as prime candidates for optimal treatment strategies in the field of regenerative medicine. Numerous studies have investigated MSCs from various sources such as human embryonic stem cells [93,94], ovarian cells [95], umbilical cord [96,97,98,99,100,101,102,103,104,105,106], placenta [70,107,108], fetal liver [109], amnion [110,111,112,113,114,115,116,117], chorionic lamina [118], menstrual blood (endometrium) [119,120,121,122,123,124] and bone marrow [125,126,127,128]. MSCs derived from human umbilical cord blood in particular have been used most frequently in animal models. MSCs improve ovarian activity and combat premature ovarian insufficiency primarily through paracrine mechanisms [108]. They release a variety of cytokines, microRNAs and other targeted agents via extracellular vesicles, leading to activation of the paracrine pathway [106,125,129]. Growth factors such as transforming growth factor-β (TGF-β), nerve growth factor (NGF), nerve growth factor receptor (TrkA), epidermal growth factor (EGF), hepatocyte growth factor (HGF), fibroblast growth factor 2 (FGF2), insulin-like growth factor (IGF-1) and vascular endothelial growth factor (VEGF) are among the secreted factors [114,130]. Some of the TGF-β superfamily proteins act like key cell receptors on granulosa cells and activate preovulatory follicular development [131,132].

Recent studies suggest that NGF is involved in the integrity of ovarian sympathetic innervation [133] as well as follicle formation and initiation of folliculogenesis [134]. Deficiency of NGF enhances the expression of p75 and TrkA, which interferes with the subsequent development of primordial follicles [134,135]. The EGF signaling pathway downregulates the somatic signal 3′5′-cyclic guanine monophosphate, which suppresses oocyte meiotic maturation while triggering meiotic signaling. EGF and its complex signaling network also control the translation of maternal transcripts in the quiescent oocyte, a process essential for oocyte competence [136]. FGF2 produced by the cumulus cells activates an autocrine/paracrine FGF2/FGFR loop within the cumulus–oocyte complex to promote oocyte maturation and meiosis [137]. IGF-1 acts through the PI3K/AKT pathway and regulates downstream proteins that may improve ovarian function [138,139]. Thus, growth factors secreted by stem cells can regulate ovarian function and follicular development.

### 3.2. Involvement of MicroRNA

MicroRNAs (miR) also contribute to the paracrine effects of MSCs. For example, miR-21 has been shown to enhance the therapeutic effect of MSCs at POI by reducing granulosa cell apoptosis through targeting PTEN and programmed cell death protein 4 (PDCD4). In a landmark study, D.M. Pegtel [140] reported that exosomes derived from human amniotic mesenchymal stem cells (hAMSC exos) showed a remarkable anti-apoptotic effect in granulosa cells (hGCs) and human ovaries undergoing premature failure due to chemotherapy [141]. Ding and colleagues showed that hAMSC-derived exosomal miR-320 inhibited hGC apoptosis and decreased the expression of SIRT4, which functions as a central sensor of cellular energy status and mitochondrial dynamics. In Ji’s work, the authors examined the effect of salidroside, a phenylpropanoid glycoside compound extracted from *Rhodiola rosea*, on dihydrotestosterone-induced granulose-like tumor cell lines. They found that salidroside had an inhibitory effect on cell oxidative stress and apoptosis mediated by AMPK-dependent Nrf2 activation, another essential factor responsible for mitochondrial biogenesis [142,143,144]. Aberrations in genes involved in mitochondrial fusion and structure can lead to POI [144]. The expression levels of adenine nucleotide translocase 2 (ANT2), AMP-activated protein kinase (AMPK) and long optic atrophy protein 1 (L-OPA1) were found to be increased in granulosa lutein cells and ovaries of patients diagnosed with POI. Interestingly, treatment with hAMSC exos significantly reduced the expression of SIRT4, AMPK, ANT2 and L-OPA1 in both in vivo and in vitro experiments. This reduction is particularly important because an increased SIRT4-OPA1 axis is associated with the phenomenon of age-related mitochondrial dysfunction [145]. Moreover, the study of Ding et al. identified hAMSC exosomal miR-320a as a key player in conferring resistance to ovarian senescence. The therapeutic effects were mediated through exosomal miR-320a and its impact on SIRT4 signaling, which was demonstrated by decreased ROS levels, improved ovarian weight and litter size [142].

### 3.3. PI3K-AKT Pathway

One possible molecular mechanism underlying the therapeutic effects of stem cells is the activation of the PI3K signaling pathway. Research by Jiao W. has shown that MSCs from both the umbilical cord and their conditioned medium are able to reverse follicular loss by activating the PI3K-AKT pathway [146]. Lu X. performed a study in which transplantation of human MSC resulted in a significant increase in the number of normal (non-atretic) follicles, associated with a significant decrease in the number of atretic (degenerating) follicles. In a rat model of POI induced with cisplatin treatment, transplantation of human MSC resulted in decreased cell apoptosis and improved ovarian function. This was evidenced with histological examination of the follicle number and measurement of serum levels of follicle-stimulating hormone (FSH), luteinizing hormone (LH) and estradiol (E2). This beneficial effect was attributed to the inhibition of apoptosis of interstitial theca cells by reducing autophagy and protecting granulosa cells from apoptosis. The probable biological mechanism underlying this phenomenon involves the inhibition of autophagy via the AMPK/mTOR pathway [96].

In the study conducted by P. Feng, the expression of COL6A5 and COL9A2, two downstream molecules involved in the FAK/AKT signaling pathway, was observed [119]. The COL6A5 and COL9A2 molecules are known to form a microfibrillar network that acts as a collagen “skeleton”. After treatment with cyclophosphamide, the expression of these molecules was downregulated, suggesting a destructive effect of cyclophosphamide on ovarian structure and function. However, transplantation of MSC from menstrual blood restored the expression of COL6A5 and COL9A2. This restoration resulted in an improvement in ovarian status through the ECM-dependent FAK/AKT pathway. In addition to increasing the expression of COL6A5 and COL9A2, activation of AKT would likely inhibit their targets: NR4A1, which is involved in cell survival or apoptosis, DNA repair and tumorigenesis [147,148,149], and CDKN1A, which is involved in cell cycle arrest [150,151].

In addition, the suppression of inflammatory responses plays a central role in stem cell therapy. Transplantation of human amniotic epithelial cells has been shown to be effective in reducing inflammation and inhibiting TNFα-mediated apoptosis, which in turn leads to suppression of granulosa cell apoptosis [152]. Despite the limited number of clinical trials, there is evidence that transplantation of human-umbilical-cord-derived MSCs can restore ovarian function in animal models of chemotherapy-induced POI, primarily through paracrine secretion rather than differentiation into oocytes or granulosa cells [106,125,129].

## 4. Extracellular Vesicles

### 4.1. The Nanoparticles of Cellular Communication

EVs are particles naturally released from the cell that are delimited by a lipid bilayer and cannot replicate, i.e., do not contain a functional nucleus [153]. Recent scientific studies have drawn attention to the significant paracrine effects of stem cells mediated by EVs [154]. These studies have shed light on the role of EVs in cellular communication and the potential therapeutic applications of stem cells. The concept of extracellular vesicles was first described in 1967 by Peter Wolf, who referred to them as “platelet debris” found in blood plasma [155]. Wolf observed the presence of phospholipids and coagulation factor III on the surface of these vesicles, suggesting their possible involvement in blood coagulation. This early observation provided valuable insights into the composition and possible functions of extracellular vesicles. In subsequent years, between 1983 and 1985, exosomes, a particular type of extracellular vesicle, were formally identified [156,157,158]. To advance the field, the International Society for Extracellular Vesicles (ISEV) has made significant progress in standardizing research in this area. The society has published guidelines called “Minimal Information for Studies of Extracellular Vesicles” (MISEV) [158]. These guidelines provide a set of recommendations that researchers should follow to ensure accurate and reproducible characterization and reporting of extracellular vesicles in their studies.

### 4.2. EVs’ Markers and Characteristics

**Figure 1:**
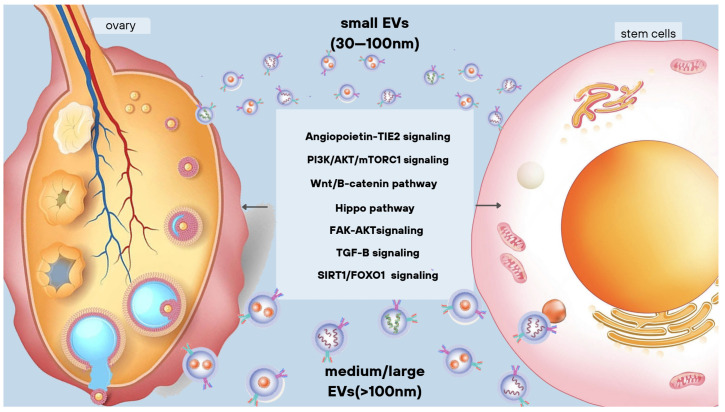
Types of extracellular vesicles and their signaling pathways.

Central to these guidelines is a classification system for EVs based on their physical and biochemical properties such as size, density, presence of lipid bilayers and expression of various surface markers (such as CD63+, CD81+, Annexin A5, etc.), as well as their source and conditions of formation (podocyte EVs, hypoxic EVs, large oncosomes, etc.). Within this framework, particles are divided into two main classes based on their size (Figure 1). The first is “small vesicles” (ranging from 20 to 200 nm), which include exomers (≤50 nm) and supermeres (≥25 nm) without lipid bilayers, as well as exosomes (40–150 nm) and defensosomes (about 80 nm). The second class, “large vesicles” (with a diameter > 200 nm), includes microvesicles (100 nm–1 µm) (MVs), migrasomes (500–3000 nm), apoptotic bodies (50 nm–5 µm) and large oncosomes (1–10 µm). This categorization brings clarity to a complex field and helps researchers to describe various factors that may influence the role of EVs in cell-to-cell communication, disease processes and therapeutic interventions [159]. This underlines the need for standardized methodologies in EV research to ensure reliable, reproducible and meaningful results.

The cargo loaded in EVs includes a variety of biomolecules, including proteins, mRNAs, non-coding RNAs, DNA, various carbohydrates and other bioactive substances [160,161]. In particular, EVs have sometimes been found to contain organelles such as mitochondria or their components [162]. Among these cargoes, proteins and miRNAs are thought to play an important role in mediating the biological effects of EVs. Interest in stem-cell-derived EVs, particularly exosomes, stems from their role as crucial mediators of paracrine effects that deliver bioactive molecules to recipient cells. This understanding opens new avenues for therapeutic interventions and drug delivery systems and provides insights into the complex interplay between stem cells and their microenvironment. Compared to stem cells, stem-cell-derived EVs possess advantages like lower immunogenicity [145,163], making them a safer option for regenerative medicine with lower immunological risks [164].

The most intensively studied type of EVs are undoubtedly small EVs, thought to originate from endosomes and often referred to as “exosomes”, which have received much attention from researchers in recent years. Exosomes have become the subject of intense scientific investigation due to their important role in intercellular communication and their potential therapeutic applications. They are released by different cell types, including stem cells, immune cells and certain cancer cells. Exosomes are rich in various biomolecules, including mRNA, long non-coding RNA (lncRNA), microRNAs, proteins and lipids [161,162]. Several biomarkers such as annexins, Rabs, TSG101, CD63, CD81, CD9, ALIX and Hsp70 are frequently found on the surface of exosomes [145,163,164]. Through this transfer of molecular cargo, exosomes can influence various physiological and pathological processes, making them an interesting field of research. Exosomes are of particular interest to researchers because of their potential as therapeutic agents and diagnostic tools. Stem-cell-derived exosomes, for example, have shown promising regenerative properties in preclinical and clinical studies [165]. They can promote tissue repair, modulate immune responses and enhance the delivery of therapeutic molecules. In addition, exosomes have gained attention as a non-invasive source of biomarkers for disease diagnoses and monitoring, as their specific cargo reflects the physiological or pathological state of the cell or tissue of origin [166]. However, it is important to note that although exosomes have been the most extensively studied, other types of extracellular vesicles, such as MVs and apoptotic bodies, also play a crucial role in intercellular communication and have their own unique properties.

### 4.3. MVs and MSCs

MVs are formed by cleavage from the cytoplasmic membrane and occupy an intermediate position between exosomes and larger vesicles in terms of size. MVs play a critical role in intercellular communication by delivering proteins, mRNA and bioactive lipids to target cells via surface-expressed ligands and surface receptors [167]. The cargo carried by MVs can influence the phenotype and function of the target cells. MVs express a variety of transmembrane proteins that contribute to their identification and characterization. Some of the commonly used transmembrane proteins as markers for MVs include CD9, CD63, CD81, TSG101, HSP70 and the protein associated with the multivesicular biosynthesis-related protein ALIX [168,169,170,171,172,173,174]. It is important to note that exosomes can also express similar proteins, highlighting the need for careful characterization and differentiation between different types of extracellular vesicles. The MVs released by MSCs have remarkable effects on tissue repair. They have shown the ability to stimulate tissue repair both in vitro and in vivo, possibly through a process of horizontal transfer of mRNA and growth factors [85,175], highlighting the significant role that MSC-derived MVs may play in mediating paracrine effects and supporting regenerative processes.

In the context of stem cell therapy for POI, clinical trials involving transplantation of MSCs have shown promising results in restoring ovarian function. Clinical trials in this area are still limited, but there is evidence that MSC transplantation may have a positive impact on the recovery of ovarian function in POI patients. Table 1 provides a comprehensive overview of the ongoing clinical trials in this area, highlighting the potential therapeutic applications and current advances in stem cell therapy for POI. Among the completed clinical trials, the work of Ding and colleagues is noteworthy (NCT02644447) [176]. They demonstrated that mesenchymal stem cells derived from the umbilical cord on a collagen scaffold promote long-term restoration of ovarian function, including ovarian blood flow, estradiol levels and follicular development, and improve overall fertility in women with POI. Another clinical trial (NCT02696889), the results of which were published by Igboeli and colleagues [177], showed that the administration of autologous bone marrow stem cells to the ovaries reduced the menopausal symptoms observed during preoperative assessments and treated patients returned to menstruation within 7 months of MSC transplantation, and no complications or safety concerns were reported. Information on the results of the remaining completed clinical trials has not yet been published.

## 5. Therapeutic Effects of Extracellular Vesicles 

### 5.1. MSC-Derived Extracellular Vesicles

EVs and their cargo of microRNAs may mediate communication between stem cells and ovarian cells and potentially influence cellular processes, including apoptosis and hormone regulation. In 2017, the use of EVs for the treatment of POI was first described. The effects of human MSC-derived exosomes on cisplatin-induced apoptosis of OGC were studied in vitro. The exosomes were successfully isolated from the supernatant of MSC cultures, and their uptake by OGC was observed with fluorescence staining. A flow cytometry analysis showed an increase in the number of viable OGC after treatment with exosomes. It was predicted that microRNAs detected in exosomes such as microRNA-24, microRNA-106a, microRNA-19b and microRNA-25 were closely associated with an anti-apoptosis effect [166]. These results suggest that MSC-derived exosomes can effectively protect OGCs from cisplatin-induced injury in vitro, highlighting their potential therapeutic application in the prevention and treatment of chemotherapy-induced ovarian damage.

The therapeutic benefit of EVs is based on their influence on both the HIPPO signaling pathway and the PI3K-AKT pathway [169,176,178]. Yang et al. further demonstrated that EVs could activate the PI3K-AKT pathway and promote VEGF expression, contributing to the improvement of angiogenesis and possible recovery in injured ovaries [168]. This study investigated the potential of MVs released from human umbilical cord MSCs to restore ovarian function in a chemotherapy-induced POI model. After transplantation, MVs were observed in the ovarian tissue and were found to migrate into the follicles within 24 h, resulting in an increase in body weight, number of ovarian follicles, induction of ovarian angiogenesis, and normalization of the estrous cycle in the POI mice. This finding suggests that MVs’ transplantation induced angiogenesis and promoted the formation of new blood vessels in the chemotherapy-injured ovaries. MVs derived from a human umbilical cord contain numerous angiogenesis-promoting biomolecules, including angiogenin, VEGF, VEGF-R2 and MCP-1 [179], some of which are present at higher levels in MVs than in the parent cells. Exosomes of MSC also contain a wide range of proteins capable of regulating AKT and Wnt signaling pathways critical for modulating ovarian function [180].

A number of microRNAs contained in extracellular vesicles, including miR-126-3p, miR-21, miR-29a and miR-17-5P, are involved in post-transcriptional regulation of gene expression and may influence ovarian function and folliculogenesis [164,171,172,173]. MiR-21 and its downstream targets Large tumor suppressor kinase 1 (LATS1), Lysyl oxidase like 2 (LOXL2) and Yes-associated protein 1 (YAP1) are thought to play an important role in the regulation of estrogen secretion and granulosa cell proliferation, both key processes in female reproductive physiology. One mechanism by which this may occur is through modulation of the transcriptional program of ERα, the primary estrogen receptor. Specifically, miR-21 targets are thought to block ERα activity through downstream signaling interactions. YAP1, a potent effector in the Hippo signaling pathway, is an important element in this mechanism. For example, in certain cellular contexts, YAP is able to translocate to the nucleus where it competes with ERα for binding to TEAD transcription factors, a group of proteins involved in cell development and growth. This competitive action leads to the dissociation of ERα from its complex, resulting in the degradation of ERα and the subsequent disruption of its function in mediating estrogen responses [172,181].

MSC-derived exosomes enriched in miR-21 have been shown to promote estrogen secretion in ovarian granulosa cells. This effect was associated with a downregulation of LATS1, which reduced the phosphorylation of LOXL2 and YAP. These results suggest the potential of MSC-derived exosomes as a treatment strategy for premature ovarian failure [171].

In the study by Gao [170], exosomes from human umbilical cord MSCs carrying upregulated miR-29a were found to promote the proliferation of GCs and inhibit their apoptosis both in vitro and in vivo. The upregulation of miR-29a also contributed to maintaining mature follicles and compensating for the decrease in AMH and E2 levels, as well as restoring the increase in FSH levels. Mechanistically, miR-29a acts on HBP1 and negatively regulates its expression. The downregulation of HBP1 promotes the activation of Wnt/β-catenin, which is involved in the regulation of ovarian functions. Moreover, upregulation of HBP1 reversed the suppressive effects of miR-29a upregulation on granulosa cell apoptosis and inactivated the Wnt/β-catenin pathway [172].

Recently, new insights were gained with a study showing that miR-126-3p was demonstrated to be a key regulatory factor in GCs. Specifically, miR-126-3p was found to promote the proliferation of ovarian GCs while inhibiting their apoptosis. The mechanisms involving miR-126-3p in this modulation emphasize the involvement of the PI3K/AKT/mTOR signaling pathway. It was discovered that miR-126-3p activates this pathway by interacting with regulatory subunit 2 of phosphoinositide 3-kinase (PIK3R2), a gene identified as a target of miR-126-3p [182]. This highlights the complex relationships between miRNAs, intracellular signaling pathways and the regulation of cell proliferation and apoptosis.

In the research conducted by E. Geherson, transplantation of exosomes derived from human umbilical cord MSCs resulted in the restoration of hormone levels associated with ovarian function and the number of ovarian follicles returned to near-normal levels. Specific mRNAs have been identified that can regulate key molecules involved in the Hippo pathway, which play a critical role in folliculogenesis and ovarian function by regulating follicle activation, ovarian cell survival and proliferation [183]. Key molecules of the Hippo pathway, including LATS1/2, MST1/2 (mammalian STE20-like kinases), YAP, TAZ and TEAD (TEA domain transcription factor), have been found to have a significant regulatory function in the cytoplasm of granulosa cells [88,184,185]. In a recent study, miR-145-5p carried by hUCMSC-EVs was confirmed to attenuate granulosa cell oxidative damage and apoptosis, leading to improved ovarian function in POI rats [186].

### 5.2. Amniotic Fluid Stem Cells as a Source of EVs

Amniotic fluid, the protective fluid surrounding an unborn child in utero, is a rich and complex biological material that can be a source of EVs. Amniotic fluid contains a diverse population of cells, including amniotic fluid stem cells (AFSCs), fetal cells and extracellular vesicles secreted by these cells. The EVs present in amniotic fluid are mainly derived from fetal urine and lung secretions, but also from AFSCs. Amniotic-fluid-derived EVs are gaining interest for potential therapeutic application, as they are thought to have several beneficial properties that could be used in regenerative medicine, tissue engineering and disease treatment [187]. To date, there have been no direct studies on extracellular vesicles isolated from amniotic fluid for the treatment of ovarian disorders, such as those occurring after a cesarean section. However, research suggests that AFSC-derived exosomes show promise for the treatment of chemotherapy-induced premature ovarian failure by preventing follicular atresia through their anti-apoptotic effect on damaged GCs. These exosomes carry microRNAs, including miR-146a, miR-10a and miR-369-3p, which are highly concentrated and target genes critical for the regulation of apoptosis. The therapeutic potential of miR-10a and miR-369-3p has been demonstrated in preclinical POF mouse models [188,189].

### 5.3. Menstrual-Blood-Derived Stem Cells as a Source of EVs

Menstrual blood represents a potential non-invasive source of stem cells and EVs that can be used to treat reproductive disorders. Menstrual-blood-derived stem cells (MenSCs) exhibit characteristics of MSCs and have the ability to differentiate into multiple developmental lineages, offering potential for the regeneration and repair of damaged tissue [190]. Preliminary research suggests that both MenSCs and their derived EVs have immunomodulatory, anti-inflammatory and regenerative properties [191]. In addition, the use of menstrual blood offers the advantage of accessibility, as it can be collected non-invasively and regularly, expanding the potential for ongoing studies and therapeutic applications. Recent studies, both in vitro and in vivo, have demonstrated the beneficial effects of MenSC-derived EVs in the treatment of premature POI. This study investigated the effects of exosomes on ovarian culture development in vitro. It was found that exposure to MenSC-derived EVs promoted the proliferation of GCs in primordial and primary follicles and increased the expression of the early follicle markers DAZL and FOXL2 while inhibiting follicular apoptosis. In an in vivo rat model POI, transplantation of MenSCs-EVs promoted follicular development, restored estrus cyclicity, regulated the ovarian extracellular matrix, recruited quiescent follicles earlier and normalized serum sex hormone levels. As a result, MenSCs-EVs significantly improved live birth outcomes, demonstrating their potential to effectively restore fertility under POI conditions [192].

It is necessary to point out the possibility of improving the embryological stage of IVF programs by co-culturing embryos with extracellular vesicles. Several studies have shown the promising potential of this approach. In a study by Marinaro et al., small EVs from MenSCs were found to improve both the quality and quantity of embryos in an aged mouse model. This effect was achieved by regulating antioxidant enzymes and promoting pluripotent activity [192]. Extracellular vesicles from the follicular fluid of young healthy women could also be an important source of extracellular vesicles. A study by Sang et al. identified microRNAs present in both MVs and the supernatant of human follicular fluid. Among these, microRNA-24 was found to regulate estradiol and progesterone concentrations, indicating its role in reproductive, endocrine and metabolic processes [193]. The potential use of EVs from the follicular fluid (ffEVs) of mares for in vitro maturation of oocytes has been demonstrated. Coculture of ffEVs during a two-step maturation method resulted in a remarkable increase in the maturation rate of compacted cumulus–oocyte complexes. These results highlight the potential of incorporating ffEVs into culture media as a promising approach to improve the effectiveness of in vitro maturation [194]. These studies suggest that transplantation of vesicles from healthy young donors into the ovaries of women with POI may be clinically promising, but this still requires experimental testing in model systems.

## 6. Discussion and the Prospect of Translation into Clinical Practice

POI is a multifaceted disease with many pathogenetic and molecular factors. The intricate landscape of ovarian dynamics and the challenges posed by conditions like POI have spurred a lot of scientific inquiry. Stem cell therapy, particularly the use of MSCs and their EVs, has emerged as a promising trend in restoring ovarian function and alleviating the burden of POI.

There is not yet much data on the direct clinical use of EVs. Clinical trials using MSCs have not yet been completed or their data have not been published. However, the available results indicate a marked improvement in ovarian function and a pronounced anti-apoptotic effect after exposure to MSCs or their EVs’ derivatives. It should also be noted that there are low risks and an almost complete absence of side effects when using cell therapy for POI. Through intricate cellular interactions, EVs activate paracrine mechanisms that act via microRNAs and bioactive molecules. Being in the ovary, extracellular vesicles have the potential to suppress apoptosis, stimulate angiogenesis, reduce ROS levels, impart resistance to ovarian aging and awaken the regenerative potential latent within, taking part in the regulation of HIPPO and PI3K-AKT signaling pathways.

MicroRNAs such as miR-21, miR-29a and miR-126-3p guide estrogen secretion, cellular proliferation and apoptosis resistance. Present findings collectively highlight the potential therapeutic significance of extracellular vesicles and their encapsulated mRNAs derived from MSCs in the context of ovarian function and granulosa cell health. The modulation of specific mRNAs encapsulated in these vesicles may offer promising avenues for the development of novel therapeutic strategies to treat ovarian-related disorders and promote reproductive health.

## 7. Conclusions

The information on the underlying molecular mechanisms involved in the therapeutic effects of EVs suggests that the manipulation and application of extracellular vesicles may hold promise for the treatment of ovarian disorders and infertility. Further research and a better understanding of the molecular mechanisms involved in the action of extracellular vesicles are essential to fully exploit their therapeutic potential. 

## Figures and Tables

**Table 1:** Review of clinical studies investigating the use of stem cells and their extracellular vesicles for the treatment of POI according to clinicaltrials.gov.

Stem Cell Therapy	Name of the Clinical Study	Link to Clinical Study	Status	Country
Autologous bone marrow transplant	Autologous bone marrow transplantation for POI	NCT02779374	Unknown	Egypt
Autologous mesenchymal bone marrow stem cells	Autologous bone marrow stem cell transplantation in patients with POI	NCT03069209	Active	Jordan
Intraovarian injection of adipose-derived stromal cells	Transplantation of autologous adipose-derived stem cells in patients with POI	NCT02603744	Unknown	Iran
Stem cells derived from bone marrow	Restoring the reserve in patients with POI with stem cells	NCT02696889	Active	USA
Mesenchymal stem cells derived from the umbilical cord	Transplantation of mesenchymal stem cells derived from the umbilical cord, with the introduction of collagen in patients with POI	NCT02644447	Completed	China
Autologous mesenchymal bone marrow stem cells	Autologous mesenchymal stem cell transplantation in women with POI	NCT02062931	Unknown	Egypt
Transplantation of autologous bone marrow stem cells	Autologous stem cell ovarian transplantation	NCT02240342	Unknown	Spain
Autologous Very Small Embryonic-Like Stem Cells	Very Small Embryonic-Like Stem Cells for ovary	NCT03985462	Withdrawn	China
Transplantation of Human Umbilical Cord Blood Mononuclear Cells and human umbilical cord mesenchymal stem cells with hormone replacement therapy	Stem-cell-therapy-combined hormone replacement therapy in patients with premature ovarian failure	NCT01742533	Unknown	China
Stem cell therapy	Stem cell therapy and growth factor ovarian in vitro activation (SEGOVA)	NCT04009473	Unknown	MaltaSerbiaNorth Macedonia
Transplantation of UCA-PSC plus hormone replacement treatment (HRT) (UCA-PSC group)/transplantation of WJ-MSC plus HRT (collagen/WJ-MSC group)	Effects of UCA-PSCs in women with POF	NCT05138367	Completed	China
Autologous bone marrow stem cell	Rejuvenation of premature ovarian failure with stem cells (ROSE-1)	NCT02696889	Completed	United States
Transplantation of adipose-derived stromal cells	Effects of ADSC therapy in women with POF	NCT01853501	Unknown	China
Stem cell therapy	Ovarian stem cells from women with ovarian insufficiency	NCT01702935	Completed	United States

## Data Availability

No new data were created or analyzed in this study. Data sharing is not applicable to this article.
